# Neuromodulation for Headache Management in Pregnancy

**DOI:** 10.1007/s11916-024-01344-1

**Published:** 2025-01-07

**Authors:** Liza Smirnoff, Michelle Bravo, Tayina Hyppolite

**Affiliations:** Department of Neurology - Headache Division, University of Miami Health, University of Miami School of Medicine, 1120 NW 14th Street, 13th Floor, Miami, FL 33136 USA

**Keywords:** Neuromodulation, Pregnancy, Migraine, Non-pharmacological treatment, Women’s health

## Abstract

**Purpose of Review:**

Management of primary headache disorders during pregnancy is limited due to known teratogenicity or unknown safety of many currently available pharmaceutical therapies. Here, we explore the safety and efficacy of non-invasive neuromodulatory devices as another treatment modality for pregnant patients.

**Recent Findings:**

There are six FDA-cleared, non-invasive neuromodulatory devices currently available for the management of headache that include remote electrical neuromodulation (REN), noninvasive vagal nerve stimulation (nVNS), external trigeminal nerve stimulation (eTNS), single-pulse transcranial magnetic stimulation (sTMS), and external concurrent occipital and trigeminal neurostimulation (eCOT-NS).

**Summary:**

Neuromodulatory devices are a safe, effective, and well tolerated non-pharmacological option for migraine and other primary headache disorders. Although evidence of safety and tolerability use in pregnancy is limited, they may serve as a therapeutic alternative or adjunct to improve the care of our pregnant patients.

## Introduction

Migraine is the most common neurological disorder in women, affecting approximately 20% of women, with the highest burden occurring during childbearing years (between ages of 15 to 49). Despite the prevalence of migraine in women during their childbearing years and migraine having a major impact on maternal morbidity, there are limited treatment options– with many preventive and abortive medications having a known risk of teratogenicity, and others having very limited safety data [1, 2]. Fluctuations in estrogen, particularly during childbearing years, are known to impact migraine severity and frequency [3]. Although migraine typically improves in 2/3rds of patients during later parts of pregnancy due to hormone stabilization, there is a significant percentage of women who may still experience worsening of their migraine disorder, especially in the first trimester [3, 4]. Other headache disorders likewise continue throughout pregnancy, requiring continued care, and safer therapeutic alternatives.

Many pharmacological options are contraindicated during pregnancy, limiting treatment options. In fact, many women with migraine or other headache disorders chose to delay or forgo family planning due to limited treatment options [5]. Furthermore pregnant women are largely excluded from clinical research trials, further limiting our knowledge and treatment options as many currently used medications for migraine have unknown teratogenicity risk (FDA Category C). Further study is needed into the scientific and ethical considerations in the inclusion of women in clinical trials [6]. The FDA has provided guidelines on pregnancy-risk categories to grade the safety of medications during pregnancy, as outlined in Table 1. Although the FDA has released a new system entitled the Pregnancy and Lactation Labeling Rule, the former guideline is still the most commonly used [7].

**Table 1:** FDA Pregnancy Risk Categories

Category	Description
A	No risk in human studies (during first trimester)
B	No risk in animal studies (no adequate studies in humans)
C	Risk cannot be ruled out (no studies in humans, animal studies show risk to fetus)
D	Evidence of risk (risk to fetus in human studies, but potential benefits of medication may outweigh risks)
X	Contraindicated (high risk to fetus in human and animal studies, risks of drug outweigh benefits)

In terms of pharmacologic abortive treatment options, acetaminophen is traditionally thought to be a safe treatment option, however, efficacy in achieving pain relief and/or pain freedom can be limited, with some recent studies suggesting a possible association between acetaminophen use in pregnancy and childhood attention-deficit/hyperactivity disorder (ADHD) [8]. Triptans have higher efficacy levels for the acute treatment of a migraine attack, however there is limited safety data during pregnancy, with one meta-analysis noting a significant increase in the rates of spontaneous abortions [1, 9]. Furthermore, there is limited safety data on the use of calcitonin gene-related peptide (CGRP) receptor-blocking agents during pregnancy. Previous studies looking at pre-eclampsia have shown abnormal CGRP response, as well as animal studies suggesting a relationship between CGRP response and fetal growth restriction [10, 11].

Of migraine prophylaxis therapies, beta blockers may cause neonatal bradycardia, hypotension, and hypoglycemia in the third trimester [12]. Angiotensin-converting enzyme (ACE) inhibitors and Angiotensin-2 receptor blockers are known to cause congenital malformations, as well as venlafaxine and tricyclic antidepressants [13]. Antiseizure medications such as valproate, are known to cause neural tube defects, cardiac defects, cleft palate defects, and exposure in-utero is associated with lower IQ scores [12]. Topiramate is associated with cleft palate/lip deformities and low birth weight, while lamotrigine may cause an increased risk of autism/dyspraxia. CGRP blocking monoclonal antibodies have limited safety data during pregnancy, but are currently not recommended due to fetal restriction in animal studies and human pre-eclampsia studies showing decreased CGRP activity [11]. Data for onabotulinumtoxinA is limited, but nerve blocks with lidocaine are considered largely safe in pregnancy [14, 15].

There are six FDA-cleared, non-invasive neuromodulatory devices currently available for the management of headache that include remote electrical neuromodulation (REN), noninvasive vagal nerve stimulation (nVNS), external trigeminal nerve stimulation (eTNS), single-pulse transcranial magnetic stimulation (sTMS), and external concurrent occipital and trigeminal neurostimulation (eCOT-NS) [16]. Although data on the safety of these neuromodulatory devices is limited, there is growing data to support the efficacy of these devices, which in many cases is comparable to pharmacological management (Table 2). Use of these devices is currently an emerging modality for the acute and preventive treatment of migraine, particularly in patients in whom a non-pharmacological option may be desired, or have medical contraindications to pharmacologic therapies, such as in pregnancy. In this review, we describe the data regarding the efficacy and safety of these devices as well as any potentially known safety data in pregnancy.

**Table 2:** Non-Invasive Neuromodulation Devices FDA-Cleared for use in Primary Headache Disorders

	Remote electrical neuromodulation (REN)	Noninvasive vagus nerve stimulation (nVNS)	Single-pulse transcranial magnetic stimulation (sTMS)	External trigeminal nerve stimulation (eTNS)	External concurrent occipital and trigeminal nerve stimulation (eCOT-NS)	External trigeminal nerve stimulation (eTNS)
Brand Name	Nerivio®	GammaCore™	SAVI dual™, eNeura Inc. SpringTMS®	Cefaly®	Relivion®	HeadaTerm1, HeadaTerm2
Cost (as of 06/2024)	$49 for 18 uses first device, afterwards $89 per device	$600 every 3 months	$395 monthly subscription	$421+ one-time cost	$150 for 60 days, total $650 after, or $75 per month	HeadaTerm1: $49 one-time cost HeadaTerm2: $99.99 one-time cost
Device Wearability	Armband adhesive (latex- free)	Handheld, used with electroconductive gel (depending on the brand, may or may not contain latex)	Handheld; no adhesive	Forehead adhesive (latex free)	Headband with water-based electrodes	Forehead adhesive (non-latex)
Stimulation Type	Electric	Electric	Magnetic	Electric	Electric	Electric
Target and Therapeutic Effect	Nociceptive receptors of the upper arm Induction of conditioned pain modulation (CPM) in the brainstem via peripheral nerves	Vagus nerve (right or left) Modulation of the autonomic nervous system, inhibition of cortical spreading depression, and alteration of nociceptive trigeminovascular neurotransmission, as well as descending pain pathways [28–30]	Occiput Modulates cortical spreading depression, neurotransmission of GABAergic circuits, and thalamocortical activity [50, 51]	Ophthalmic Nerve (V1)- Supraorbital and Supratrochlear Branches Therapeutic effect is unclear	Supraorbital and supratrochlear nerves, greater occipital nerves Neurotransmission of trigeminal and occipital inputs to the trigeminocervical complex in the brainstem [70]	Ophthalmic Nerve (V1)- Supraorbital and Supratrochlear Branches Therapeutic effect is unclear
Mechanism of Action	Electrical signal of a symmetrical biphasic square pulse with a modulated frequency of 100‐120 Hz, a pulse width of 400 μs, and an output current up to 40 mA [17]	Electrical signal comprising a 5-kHz sine wave burst lasting for 1 ms (5 sine waves, each lasting 200 μs), with such bursts repeated once every 40 ms (25 Hz), generating a 24-V peak voltage and 60-mA peak output current	Single pulse of magnetic stimulation	Rectangular biphasic compensated impulses with an electrical mean equal to zero, 250 µS impulse width, 60 Hz frequency, maximum intensity of 16 mA with a progressive slope from 1 to 16 mA over 14 min	Electrical signal comprising a phase width 100 [micro]s, a pulse frequency 0.33 Hz, trigeminal stimulation intensity up to 5 mA, and occipital stimulation intensity up to 7 mA	Pulse repetition frequency of 50 Hz, a pulse width of 125 μs, and an impulse amplitude of 60 V
FDA Indication(s) for Adults	Acute treatment of migraine for ages 12 + [17, 21] Preventive treatment of EM and CM [20, 74]	EM preventive, acute [31, 32] cCH preventive [37] eCH acute [36] HC and PH acute [43, 44]	Preventive treatment of migraine [54] Acute treatment of migraine [52]	Preventive treatment of migraine [66] Acute treatment of migraine [64, 65]	EM acute [70] CM acute [70]	Acute treatment of migraine [68]
Adverse Effects	Paraesthesias and dysesthesias, warmth, muscle spasms, temporary numbness, pain in the arm, shoulders or neck	Application site discomfort Muscle twitching, Pain in face, head, teeth	Transient light headedness Scalp discomfort or tingling Tinnitus Dizziness	Stimulation-induced paresthesia or dysesthesias Sleepiness Skin irritation Nausea	Paraesthesias and dysesthesias, temporary numbness, skin irritation and erythema,	None listed
Safety Considerations	Not evaluated in persons with congestive heart failure, severe cardiac or cerebrovascular disease Should be applied over dry, healthy skin with normal physical sensation and without any metallic implants or in proximity to cancerous lesions	Not evaluated in patients with carotid artery atherosclerosis, cervical vagotomy, clinically significant hyper/hypotension or brady/tachycardia Not evaluated in patients with metallic devices implanted at/near neck (stent, bone plate/screw)	Long-term effects of sTMS are unknown	Not evaluated in patients who have received supraorbital nerve blocks or Botox treatment in the prior 4 months	Not evaluated in patients with suspected or diagnosed heart disease or epilepsy	None listed
Contraindications	Uncontrolled Epilepsy Any active implanted electrical device	Any implanted electronic medical device Simultaneous use of another portable electronic device	Conductive metal implants in the head and neck	Metallic or electric devices implanted in the head Cardiac pacemaker or implanted or wearable defibrillator Pain of unknown origin	Metal implants or shrapnel in the head (except dental implants) Recent (< 3 months) brain or facial trauma Skin abrasions on the forehead or occiput at the contact area Implanted neurostimulators or any implanted metallic or electronic device in the head Cardiac pacemaker or an implanted or wearable defibrillator	None listed
Evidence for safety during pregnancy	Retrospective case control study of 140 women (59 REN, 81 controls) did not show a difference in pregnancy outcomes [23] Daily TENS usage on the abdomen in pregnant mice showed no teratogenic effects [24] An RCT for the use of TENS device for pregnancy-related pelvic pain in 30 patients did not reveal any negative impact during pregnancy [25] RCT of acupuncture vs. TENS device use for pelvic girdle pain during pregnancy in 113 women did not show negative related birth outcomes [26]	One retrospective review of invasive VNS in 44 pregnancies did not reveal birth defects or developmental complications [48] A cohort study looking at use invasive VNS treatment during pregnancy, showed increased obstetrical complications but no teratogenicity [49] Invasive VNS stimulation in pregnant rats 6–7 days before delivery showed that neither pup viability nor number of cells labeled for pro-inflammatory cytokines in the nucleus tractus solitarii or hypoglossal motor nucleus was impaired by VNS [46] Invasive VNS can potentially have a protective effect in pregnant rats with pre-eclampsia model [47]	Repetitive TMS for the treatment of anxiety/depression during pregnancy was not associated with poor cognitive or motor development outcomes for the fetus [57–59] A case control study of 30 pregnant patients who received rTMS vs. controls did not show a difference in motor or cognitive outcomes of their children at ages 18–62 months [57] Exposure to MRI (without contrast) during the first trimester of pregnancy was not associated with increased risk of harm to the fetus [60]	Not evaluated previously, but e-TNS device used for depression in one case [69]	Not evaluated previously, but e-TNS device used for depression in one case [69] Two cases two patients with invasive occipital stimulation during pregnancy resulted in 4 healthy pregnancies [72, 73]	Not evaluated previously, but e-TNS device used for depression in one case [69]
Level of evidence in safety during pregnancy (using Sackett criteria [75])	Level III	Level IV	Level III	Level U	Level U	Level U

Six non-invasive FDA-cleared devices for primary headache, mainly for migraine, include two devices using external trigeminal stimulation (A-B), transcranial magnetic stimulation (C) non-invasive vagal nerve stimulation (D), remote electrical neuromodulation (E) and combined trigeminal and occipital neuromodulation (F). Level of safety evidence of the neuromodulatory devices during pregnancy was calculated using Sackett criteria. Other relevant safety data was included, which encompasses other devices with similar functionality to the devices depicted above and/or used for different indications, as well as animal studies, and fetal outcomes

## Non-Invasive Neuromodulation Devices for Headache Disorders

### Remote Electrical Neuromodulation

Remote Electrical Neuromodulation (REN), or Nerivio®, was first approved by the FDA for the acute management of migraine in 2019. It is a small wearable device that is applied to the upper arm with electrodes and secured with an arm band. It is thought to exert its effects by inducing conditioned pain modulation (CPM) in the brainstem via peripheral nerves, which ultimately results in serotonergic and noradrenergic modulation of pain and associated migraine symptoms [17]. The initial pilot study conducted by Yarnitsky et al. (in 2017) was a randomized controlled trial (RCT) looking at the use of the device for the acute treatment of migraine, showed 50% pain reduction in 2 h, and a 46–48% pain reduction at two hours when the device was used at the strongest stimulations of P200 and P150 for a 20-min treatment, as compared with 26% pain improvement at two hours with a sham device [18]. Efficacy was redemonstrated in a follow-up RCT completed in 2019 amongst 126 participants and 126 controls. Participants were asked to record symptoms of pain, nausea, photophobia, and phonophobia, and to indicate their most bothersome symptom (MBS) prior to the treatment and after the 45 min treatment, which allowed the participant to modulate the intensity via an application on their smartphone [17]. 66.7% of participants in the active group and 38.8% participants in the sham group reported pain reduction of 46.3% vs. 22.2% (*P* < 0.0001), and 37.4% of participants and 18.4% of controls reported pain freedom after treatment (*P* = 0.003).

The 48-h time frame between treatments for the preventive treatment of migraine was established based on the results of the REN acute open label study conducted by Nierenberg et al. in 2020 in 38 participants, which demonstrated acute pain relief at 24 h in 45% of participants in at least 50% of attacks [19]. This study was followed up with a prospective RCT published by Tepper in 2023, with 177 patients were either randomized to the REN device or sham device with every other day use for 8 weeks [20]. This study demonstrated a monthly reduction of −3.2 versus −1.0 (*p* = 0.003) headache days in the episodic migraine group, and 4.7 vs.1.6 (*p* = 0.001) in the chronic migraine group. Since, several studies have evaluated and confirmed the safety and efficacy of the REN device in adolescents [21, 22].

To note, a recent retrospective survey study published in 2023 by Peretz et al., of 140 pregnant patients, evaluated the safety of the REN device in pregnancy, and included 59 women who used the REN device during pregnancy, and 81 controls who did not [23]. The study did not demonstrate any statistical difference between the gestational ages of the pregnancies, newborn weights, preterm births, birth defects, stillbirth births, or milestones at three months of age.

Multiple studies on the use of other transcutaneous electrical nerve stimulation (TENS) devices in pregnancy have been conducted. Animal studies have shown that daily TENS device used on the abdomen throughout pregnancy in mice showed no teratogenic effects [24]. A randomized clinical trial for the use of TENS device for pregnancy-related pelvic pain in 30 patients likewise did not reveal any negative impact during pregnancy [25]. Another randomized controlled trial of acupuncture vs. TENS device use for pelvic girdle pain during pregnancy in 113 women did not show negative related birth outcomes [26].

### Non-Invasive Vagus Nerve Stimulator (nVNS)

The non-invasive vagus nerve stimulator (nVNS), otherwise known as Gammacore™, is an FDA-cleared non-invasive vagus nerve stimulator device indicated for the prevention and acute treatment of migraine with or without aura in adolescents (12 years of age and older) and adults, as well as for the prevention and treatment of cluster headache, and the acute treatment of paroxysmal hemicrania and hemicrania continua [27]. For the prevention of migraine, two, 2-min treatments are conducted morning and night, with the device applied along the cervical branch of the vagus nerve (on the side of the neck), where it emits transcutaneous electrical impulses in a sinusodual and biphasic pattern. It is thought to exert its effects by modulating the autonomic nervous system, inhibiting cortical spreading depression, and altering nociceptive trigeminovascular neurotransmission, as well as descending pain pathways [28–30].

nVNS was cleared for the acute treatment of episodic migraine in adults based on the results of the PRESTO study published in 2018 [31]. This was a double-blinded, sham-controlled randomized clinical trial that demonstrated nVNS to be superior to sham for pain freedom at 30 min (12.7% vs 4.2%; *p* = 0.012) and 60 min (21.0% vs 10.0%; *p* = 0.023) but not at 120 min (30.4% vs 19.7%; *p* = 0.067). nVNS was later approved for the prevention of episodic migraine based on the results of PREMIUM trial (a double-blind, sham-controlled randomized control trial) published in 2019 [32]. Although the primary outcome (mean reduction in the number of migraine days) was not met, a post hoc analysis of high frequency users demonstrated a significant reduction in monthly migraine days (2.27 vs. 1.53; *p* = 0.043) with a significantly higher reduction in monthly migraine days in patients with aura (nVNS, − 2.83 days; sham, − 1.41 days; *p* = 0.061) as compared to patients without aura (nVNS, − 2.22 days; sham, − 1.71 days; *p* = 0.15) [32]. Further studies have led to the approval of the device for the prevention of migraine in the adolescent population [33].

In regard to its FDA indication for the acute treatment of episodic cluster headache, the ACT1 and ACT2 trials (both double-blinded, randomized sham-controlled trials) studied pain relief and pain freedom within 15 min [34, 35]. When the data was analyzed in a pooled fixed-effects model, nVNS was found to be superior to sham in treatment of episodic cluster headache, but not chronic cluster headache (both endpoints *p* < 0.01) [36]. The use of nVNS for the prevention and acute treatment of chronic cluster headache was studied in the PREVA trial, showing a significantly higher response rate (defined as the proportion of participants with > 50% reduction of mean number of cluster headache attacks per week during the randomized phase in the SoC plus nVNS group (40% (18/45)) than in the control group (8.3% (4/48)) (*p* < 0.001) [37]. In addition, real-world studies have shown that there was an improvement in quality of life for patients with cluster headache who did not previously respond to preventive and/or acute pharmacologic treatments [38, 39].

In review of the literature, other potential future applications of nVNS include the acute treatment of vestibular migraine, menstrual migraine and primary cough headache [40–42]. Currently, nVNS also holds an FDA indication for hemicrania continua, and paroxysmal hemicrania [43, 44].

According to the American Headache Society recommendations on treatment of migraine during pregnancy, nVNS is a tool that has been suggested for consideration [45]. There is currently no data on the safety and efficacy of nVNS during pregnancy, however there is some safety data for invasive VNS. In animal studies, pregnant rats were exposed to invasive VNS stimulation 6–7 days before delivery. After delivery, the pup brainstems were collected for further analysis. This study showed that neither pup viability nor number of cells labeled for pro-inflammatory cytokines in the nucleus tractus solitarii or hypoglossal motor nucleus was impaired by VNS [46]. Other animal studies have showed that invasive VNS can potentially have a protective effect in pregnant rats with pre-eclampsia induced by N-nitro-L-arginine methyl ester [47]. In patients who use invasive VNS for epilepsy, a comprehensive literature search of 44 patients was conducted and suggested that invasive VNS may be relatively safe for the mother and fetus, and turning off invasive VNS during pregnancy may be unnecessary [48]. Regarding maternal outcomes, 2 out of 44 women in this study had spontaneous abortions during the first trimester of pregnancy, with one case thought to be due anti-seizure medications the patient was using at the time. In terms of fetal outcomes, 1 fetus (out of 44) was born with severe fetal malformations that was attributed to the anti-seizure medications the patient was using. In another study, the International Registry of Antiepileptic Drugs and Pregnancy database was used to identify 25 women who had invasive VNS treatment during pregnancy [49]. Results suggested an increased rate of obstetrical complications, but no teratogenicity. Given the broad implications of nVNS for multiple headache disorders, more research on safety in pregnant women should be pursued.

### Single-Pulse Transcranial Magnetic Stimulation (sTMS)

The single-transcranial magnetic stimulation (sTMS) device (SAVI dual™ or eNeura Inc. SpringTMS®), is an FDA-cleared device indicated for the prevention and acute treatment of episodic migraine with or without aura in adolescents (12 years of age and older), and adults [27]. The device uses a transcranial magnetic stimulation of 0.9 Tesla, which is theorized to modulate cortical spreading depression, brain excitability, neurotransmission of GABAergic circuits, and thalamocortical activity in animal models [50, 51]. The portable device is applied to the occiput and emits a single pulse of magnetic stimulation within one second. It can be used for prevention (four pulses twice a day) or for abortive treatment of migraine (3–4 pulses at the onset of a migraine attack).

sTMS was first studied for the acute treatment of migraine in 2010 by Lipton et al. in a randomized, double-blind, parallel-group, two-phased, sham-controlled study in 18 headache centers across the US. Pain-free response rates at 2 h were significantly higher in the sTMS group (39%) as compared to sham stimulation (22%), with a therapeutic gain of 17% (95% CI 3–31%; *p* = 0.0179) [52]. The indication for sTMS for the treatment of acute migraine in adolescents 12 years of age and older was expanded in 2018, after an open-label feasibility study was conducted, demonstrating sTMS to be a feasible and well-tolerated treatment when used for prevention over the course of a month, with a significant reduction in mean headache days with no serious adverse events [53].

In the ESPOUSE study, the use of sTMS for prevention of migraine was studied in a multicenter prospective observational study in which patients with migraine were treated using the sTMS device for prevention (four pulses twice daily), as well as for acute treatment (three pulses up to three times a day for each attack) [54]. The sTMS device group had a 2.75 mean reduction in headache days as compared to placebo (0.63 days, *p* < 0.0001). The study also highlights the safety of this device, with no serious adverse events, and 29% of patients reporting mild adverse events that included lightheadedness (3.7%), tingling (3.2%), and tinnitus (3.2%). The main disadvantages of this study were the lack of sham group and the study focusing on patients with episodic migraine.

In the pregnant population, there is limited data on the safety and efficacy of sTMS for the prevention and acute treatment of migraine. In a safety review published in 2020 by Dodick et al., the studies reviewed at the time showed no adverse effects on humans [55]. In a small study in the United Kingdom in 2013, three pregnant patients used the sTMS device, with all three patients reporting pain relief and shorter attack duration with no adverse side effects [56]. Repetitive TMS use on the other hand, has more safety evidence during pregnancy, given that it may potentially be a safer alternative to treat anxiety and depression during pregnancy in lieu of psychiatric medications. In women exposed to repetitive TMS during pregnancy, TMS was not associated with poor cognitive or motor development outcomes for the fetus [57–59]. Notably a case control study of 30 pregnant patients who received rTMS vs. controls did not show a difference in motor or cognitive outcomes of their children at ages 18–62 months [57]. Likewise in a retrospective cohort study conducted in Ontario Canada, exposure to MRI (without contrast) during the first trimester of pregnancy was not associated with increased risk of harm to the fetus or in early childhood [60].

### External Trigeminal Nerve Stimulation (e-TNS)

There are currently three e-TNS devices available on the market (CEFALY®, HeadaTerm1®, HeadaTerm2®). Cefaly® is an FDA-approved device for the prevention and actue treatment of episodic migraine with or without aura in patients 18 years of age and older. HeadaTerm1® and HeadaTerm2® are currently approved for the preventive treatment of migraine in patients 18 years of age and older. These are the only devices for the management of headache that are currently available to patients without a prescription. e-TNS functions by transmitting transcutaneous biphasic electrical impulses via an electrode strip to the supratrochlear and supraorbital nerves, branches of the ophthalmic branch of the trigeminal nerve (V1). The mechanism of action of e-TNS is unclear, however whole brain BOLD-fMRI (Blood oxygenation level dependent functional MRI) suggests that this device has antinociceptive effects on the anterior cingulate cortex when used for prevention [61–63].

Cefaly® was studied for the acute treatment of migraine in the ACME trial, a double-blinded, randomized sham-controlled clinical trial of 109 patients with episodic migraine which was completed in 2019 across three US headache centers showing a 59% decrease in mean pain score in the verum group as compared to the sham group, with a 30% decrease in pain (*p* < 0.0001) [64]. In 2023, in a phase-3 clinical trial, the e-TNS device was studied amongst a large sample size of 538 patients with a history of episodic migraine (ranging between 2–8 headache days per month) for the acute treatment of migraine (across 10 headache centers in the United States). Results demonstrated a higher percentage of patients with pain freedom after 2 h in the group exposed to the true device, otherwise known as the verum group (25.5%) as compared to the sham group (18.3%; *p* = 0.043), and a resolution of most bothersome migraine-associated symptoms in the verum group (56.4%), as compared to the sham group (42.3%; *p* = 0.001) [65].

The preventive indication of Cefaly® was derived from the results of the PREMICE trial, a randomized-sham controlled clinical research study of 67 participants across five headache clinics [66]. After 3 months of daily use, there was a reduction in monthly migraine days in the device group (6.94 vs 4.88; *p* = 0.023) as compared to the sham group (6.54 vs 6.22; *p* = 0.608), but it did not meet statistical significance. However, the 50% responder rate, was significantly higher in the device group compared to the sham group (38.1% vs 12.1%). Some of the limitations of this study include low sample size, enrollment of participants with low frequency migraine attacks, and partial unblinding of the device. Amongst patients with chronic migraine, a recent prospective observation open-label study published in 2023 suggested lower efficacy, with only 16.5% of patients (4 out of 24 patients) demonstrating a > 30% reduction in total headache days and migraine days, and only a marginal improvement in headache in 42% (6 out of 24 patients) [67]. Regarding the HeadaTerm e-TNS device, it was studied for the acute treatment of migraine in the emergency department in 159 patients, using the visual analogue scale (VAS) to determine improvement of migraine after 20 min and after 120 min of use. For the verum group the VAS change from 0 to 120 min was − 65 ± 25 and for the sham group it was − 9 ± 2 (*p* < 0.001) [68].

According to the American Headache Society as of 2022, the e-TNS device was proposed as a tool to consider during pregnancy for migraine, however there are no studies published to date demonstrating safety or efficacy amongst pregnant patients [45]. However, e-TNS was studied in one patient with major depressive disorder during pregnancy who did not have negative outcomes [69].

### External Concurrent Occipital and Trigeminal Neurostimulation (Relivion®)

The external concurrent occipital and trigeminal neurostimulation device (eCOT-NS), Relivion®, was first approved by the FDA in 2021 for the management of episodic and chronic migraine [70, 71]. The device is positioned as a ring around the head, stimulating the supraorbital and supratrochlear branches of the trigeminal nerves anteriorly, and the greater occipital nerve branches posteriorly through water-based electrodes. The device is thought to exert its effects through neurotransmission of trigeminal and occipital inputs to the trigeminocervical complex in the brainstem [71].

An RCT published in 2022 by Daniel et al. included 27 participants and 28 controls who were exposed to either eCOT-NS or a sham device during an acute migraine attack and were instructed to adjust the device intensity based on physician recommendations and patient comfort [70]. Scores on the VAS were recorded prior to, and after using the device. Pain severity was reduced in 53% of participants vs. 10% of controls immediately after use (*p* = 0.0002), in 52% vs. 17% at 2 h (*p* = 0.0324), and in 71% vs. 34% at 24 h (*p* = 0.0220). The study was limited due to small sample size, and few participants with history of chronic migraine. Pregnant patients were not included in this study. Only mild side effects, including one subject experiencing a headache related to treatment, were reported.

There have been several reports of the use of invasive occipital nerve stimulation in pregnancy. It has been used for refractory hemicrania continua in a patient who had 3 pregnancies following the placement and active use of the device, as well as in a pregnant patient with chronic cluster headache [72, 73]. The two patients had a total of 4 uncomplicated pregnancies, though limited as the cases did not report on further fetal outcomes.

## Conclusions

There is a growing need for safer and more effective treatment options during pregnancy. Neuromodulation has emerged as a promising tool in the treatment of headache disorders during pregnancy. The field of neuromodulation has rapidly grown in the last decade, with six FDA-approved devices currently available. Neuromodulatory devices may prove to be a beneficial adjunctive treatment, or in some patients, can be an alternative non-pharmacological treatment option with limited side effects and interactions, and in some cases given comparable efficacy to pharmacological therapies. Given notable contraindications to many commonly used migraine medications during pregnancy, neuromodulation may serve as a safer therapeutic alternative to many commonly used abortive and preventive medications for pregnant patients.

While at this time data is limited regarding the true safety of neuromodulation in pregnancy, studies conducted thus far on the available FDA- approved devices for migraine and relevant devices with similar mechanisms of action do not appear to pose a harm to the pregnant patient or the developing fetus. Out of the six FDA approved neuromodulatory devices, REN and eTNS has the highest level of safety evidence during pregnancy (Level III- per Sackett criteria), followed by nVNS (Level IV), and the remainder of devices having limited or unknown safety data (eTNS and eCOT-NS). More research is needed to further elucidate the safety and efficacy of neuromodulation when used during pregnancy. Likewise, many neuromodulatory devices have been deemed as safe treatments by the American Headache Society, suggesting headache specialist comfort with their use in this population. Further investigation into cost-effectiveness or cost-reducing programs is also needed to ensure access across diverse socioeconomic groups.

**Table:** 

Reference (Author, ref #)	Important	Very important	One sentence explanation
Yarnitksy et al., 2019, [17]	X		Trial which shows remote electrical neurostimulation can treat migraine attacks
Tepper et al., 2023, [20]	X		Trial which establishes that remote electrical neurostimulation is effective for migraine prevention
Peretz et al. 2023 [23]		XX	Retrospective study of 140 patients which did not show increased teratogenicity of REN when used in pregnancy
Vaidya et al. 2018 [25]	X		An RCT for the use of TENS device for pregnancy-related pelvic pain in 30 patients did not reveal any negative impact during pregnancy
Tassorelli et al., 2018, #23	X		Study which showed non-invasive vagal nerve stimulation can be used as abortive treatment of migraine
Diener et al., 2019, #24	X		Trial which establishes that non-invasive vagal nerve stimulation is effective for prevention of episodic migraine
Ding et al. 2021 [48]		XX	Review of invasive VNS use in 44 pregnant patients
Sabers et al. [49]		XX	Review of invasive VNS for use in 21 pregnant patients
Dodick et al. [55]	X		A safety review of sTMS in for the management of headache
Starling et al. 2018, #15	X		Pivotal study establishes TMS is effective for migraine prevention
Lipton et al., 2010, #2	X		Study which establishes TMS as an effective acute treatment of migraine
Eryilmaz et al. 2015 [57]		XX	Case control study of rTMS in pregnancy showing that rTMS is not associated with poorer cognitive or motor development outcomes in children aged 18–62 months
Chou et al., 2019, #9	X		Trial which shows that external trigeminal neurostimulation is effective in acute migraine treatment
Schoenen et al., 2013, #10	X		Study which establishes external supra-orbital stimulation's efficacy in migraine prevention
Trevizol et al. 2015 [69]			Case of e-TNS used for the management of major depressive disease in a pregnant patient
Daniel et al., 2022, #38	X		Trial which shows external concurrent occipital and trigeminal nerve stimulation is effective for abortive treatment of migraine
De Coo et al. 2016 [73]		XX	Case of invasive occipital nerve stimulation for cluster headache in a pregnant patient
Miller et al. 2017 [72]		XX	Case of invasive occipital nerve stimulation for hemicrania continua in a pregnant patient

## Data Availability

No datasets were generated or analysed during the current study.
